# Training and intrinsic evaluation of lightweight word embeddings for the clinical domain in Spanish

**DOI:** 10.3389/frai.2022.970517

**Published:** 2022-09-21

**Authors:** Carolina Chiu, Fabián Villena, Kinan Martin, Fredy Núñez, Cecilia Besa, Jocelyn Dunstan

**Affiliations:** ^1^Department of Mathematical Engineering, FCFM, Universidad de Chile, Santiago, Chile; ^2^Center for Mathematical Modeling & CNRS IRL2807, FCFM, Universidad de Chile, Santiago, Chile; ^3^Department of Computer Sciences, FCFM, University of Chile, Santiago, Chile; ^4^Department of Computer Sciences, Massachusetts Institute of Technology, Cambridge, MA, United States; ^5^Department of Language Sciences, Pontificia Universidad Católica de Chile, Santiago, Chile; ^6^Department of Radiology, School of Medicine, Pontificia Universidad Católica de Chile, Santiago, Chile; ^7^Millenium Institute for Intelligent Healthcare Engineering, ANID, Santiago, Chile; ^8^Initiative for Data & Artificial Intelligence, FCFM, University of Chile, Santiago, Chile

**Keywords:** natural language processing, Spanish language, word embeddings, medical informatics, neural networks, intrinsic evaluation, semantic evaluation

## Abstract

Resources for Natural Language Processing (NLP) are less numerous for languages different from English. In the clinical domain, where these resources are vital for obtaining new knowledge about human health and diseases, creating new resources for the Spanish language is imperative. One of the most common approaches in NLP is word embeddings, which are dense vector representations of a word, considering the word's context. This vector representation is usually the first step in various NLP tasks, such as text classification or information extraction. Therefore, in order to enrich Spanish language NLP tools, we built a Spanish clinical corpus from waiting list diagnostic suspicions, a biomedical corpus from medical journals, and term sequences sampled from the Unified Medical Language System (UMLS). These three corpora can be used to compute word embeddings models from scratch using Word2vec and fastText algorithms. Furthermore, to validate the quality of the calculated embeddings, we adapted several evaluation datasets in English, including some tests that have not been used in Spanish to the best of our knowledge. These translations were validated by two bilingual clinicians following an *ad hoc* validation standard for the translation. Even though contextualized word embeddings nowadays receive enormous attention, their calculation and deployment require specialized hardware and giant training corpora. Our static embeddings can be used in clinical applications with limited computational resources. The validation of the intrinsic test we present here can help groups working on static and contextualized word embeddings. We are releasing the training corpus and the embeddings within this publication^1^.

## 1. Introduction

Natural Language Processing (NLP) is an intersecting field between computer science and linguistics, focusing on the machine understanding of human languages. The state-of-the-art techniques for NLP are deep learning methods, which in order to create models for language understanding, need large training corpora (Otter et al., 2021). It is known that languages different from English have less resources for NLP, particularly in specific domains (Névéol et al., 2018). The Spanish language is not an exception. Nevertheless, a vibrant clinical NLP community in Spain and Latin American countries is creating resources and models to overcome this gap.

There has been a surge of migration from paper-based clinical records to Electronic Health Records (EHR) over the last decades, increasing the data availability significantly for secondary usage (Evans, 2016). However, a large proportion of EHRs are in the form of unstructured data that does not have an underlying model, and therefore its information extraction is exceedingly challenging (Sun et al., 2018).

Text data is an essential part of the EHR because healthcare professionals, as humans, process logical decisions through language. These thoughts used for decision-making are dumped as narratives into the EHR for mnemonic and legal reasons. These logical decisions are critical to patient care because they govern diagnostic and treatment planning processes (Dalianis, 2018). Even though there are attempts to systematize information by utilizing controlled vocabularies, this normalization process takes time, needs prior training, and often leads to coding errors (Horsky et al., 2018). Thus, the clinical text is an irreplaceable part of the EHR.

There is a need for information extraction from clinical narratives, and NLP can solve the issues associated with this task. In NLP, there are specific tasks that can serve to leverage the information contained in the clinical narratives, such as text classification, named entity recognition, and machine translation, to name a few (Dalianis, 2018).

Word embeddings allude to a vectorial representation of words, storing semantic information that allows the model to associate different vectors according to the grammatical context (Goldberg, 2017). These word embeddings can be used for downstream tasks inside a deep learning architecture. In fact, the usage of such pre-trained word embeddings inside deep learning models improves performance, decreases the training time, and the number of examples needed for training (Kim, 2014; Ma and Hovy, 2016). This last advantage can be especially beneficial in the Spanish-speaking medical field because of the scarcity of available training data.

For the computation of word embeddings a training corpus is needed. For this reason, we extracted data from three sources: (1) clinical narratives from The Chilean Waiting List Corpus, which is a collection of diagnostic suspicions from the waiting list in Chilean public hospitals (Báez et al., 2020; Báez et al., 2022; Villena et al., 2021b), (2) a medical journal corpus extracted from the SciELO library, which is a collection of articles from several medical journals in Spanish (Villena et al., 2020) and (3) a corpus constructed from the Unified Medical Language System (UMLS) (Bodenreider, 2004) term graph. We computed embeddings using Word2vec and fastText algorithms and validated them using classic intrinsic evaluation tests adapted to Spanish, such as word pair similarity and semantic textual similarity. For the latter, the sentences were first translated using Google Cloud Translation API and then validated by a protocol that typifies the inadequacies in translation, and applied by two bilingual clinicians. The computed embeddings are open to the community^2^.

The paper is organized as follows. Section 2 highlights previous works relevant to our research. Section 3 describes all the steps performed to compute the results; the construction of the corpora and the computation of the word embeddings, as well as the method used for validation. Section 4 provides the results for the different intrinsic tasks. Finally, a discussion of the results and overall conclusions can be found in Sections 5 and 6, respectively.

## 2. Related work

Mikolov et al. (2013) formulated two algorithms for estimating continuous representations of words using log-linear models: continuous bag-of-words (CBOW) and continuous skip-gram (skip-gram). These two algorithms calculate a vectorial representation of a word, called a word embedding, from words in the same context. Further work in Mikolov et al. (2013) led to the creation of the Word2vec framework, capable of employing either of these algorithms to produce embeddings for the words in a corpus. This framework has since been applied to various NLP tasks, and serves as the foundation for our work in this paper.

Most work on word embedding implementations targets general-domain texts and general evaluation sets. As demonstrated in Chiu et al. (2016), these general-domain implementations often do not maintain robust performance when applied to domain-specific tasks such as biomedical text analysis, even with the use of larger corpora. As such, domain-specific resources are a necessity.

Recently, in the biomedical domain, Zhang et al. (2019) trained biomedical word embeddings for a project named BioWordVec. These embeddings are capable of employing subword information, solving the problem of recognition of rare or out-of-vocabulary (OOV) words in the training data, unlike previous traditional biomedical word embeddings. Another project presented in Chen et al. (2019), BioSentVec, similarly computed biomedical embeddings on the sentence level. However, both of these models are suited for use only in English, a language abundant in resources for NLP and in-domain corpora for biomedical tasks, unlike Spanish.

In the Spanish biomedical domain, Santiso et al. (2019) created word embeddings for negation detection in Spanish-language health records. They employed both in-domain and general-domain corpora: unannotated Electronic Health Records (EHRs) from a Spanish hospital were used as in-domain data, and the SBWCE corpus^3^ was used as general-domain data. These embeddings, however, were not intrinsically evaluated nor compared performance-wise with other embeddings, and they were not made available for use. In another work, Akhtyamova et al. (2020) used the Flair (Akbik et al., 2019) and BERT (Devlin et al., 2018) models to calculate word embeddings for the Spanish clinical domain as part of a named entity recognition (NER) task and Rojas et al. (2022) computed another Flair language model from clinical narratives in Spanish. These models utilize contextualized word embeddings that take into account the word context upon embedding calculation. While capable of producing high performance models, these contextualized word embeddings have high computational requirements and are impractical to implement under computational resource constraints, unlike static embeddings.

In Soares et al. (2019), static medical word embeddings were trained for the Spanish language using the state-of-the-art fastText (Bojanowski et al., 2016) algorithm. We compare the results of our computed word embeddings with the performance of the state-of-the-art word embeddings presented in Soares et al. (2019), using as performance metrics the intrinsic evaluation methods elaborated therein, as well as other evaluation datasets adapted from English for the first time to the Spanish language, to the best of our knowledge.

In our work, we enrich the corpus of Spanish NLP resources with novel and computationally efficient word embeddings trained for Spanish clinical use on a domain-specific Spanish clinical corpus that we constructed. We further validate the quality of our embeddings through adapting several English-language evaluation datasets, some of which have not been used in the Spanish language to the best of our knowledge, and we release our training corpus and computed embeddings to the community.

## 3. Method

Using different techniques, we gathered a large amount of text data from the medical field in the Spanish language. We scraped medical journals to obtain samples from biomedical language, synthesized new text from controlled vocabularies to augment the available data, and used actual clinical narratives to obtain samples of the medical jargon. Then we combined these corpora to compute word embedding language representations using two well-known algorithms: Word2vec (Mikolov et al., 2013) and fastText (Bojanowski et al., 2016).

All the experiments described in this section were performed on a local machine (except for the embedding computation) and were developed using the Python programming language.

### 3.1. Corpora construction

#### 3.1.1. Chilean waiting list corpus

This corpus comprises diagnostic suspicions from users of the public healthcare system waiting for their first specialty consultation. These referrals were obtained *via* Chilean Transparency Law, a government-wide initiative giving every citizen the right to request anonymized public data (Martinez et al., 2019). We got access to 11,826,843 referrals collected by the authors (Villena et al., 2021b), and a subset of this corpus has been annotated with entities and relations clinically relevant^4^. These diagnostic suspicions were written directly by general practitioners in a primary-care setting. The description of this corpus is in Table 1.

**Table 1:** Corpora descriptive statistics.

**Corpus**	**Sentences**	**Tokens**	**Vocabulary**
Chilean waiting list	6,413,083	23,092,135	152,092
Medical journals	500,828	13,520,323	141,319
UMLS heading sequences	942,346	198,800,232	84,189
Total	7,856,257	235,412,690	310,117

#### 3.1.2. Medical journals corpus

It comprises Spanish medical articles^5^ extracted from the SciELO website. It was constructed using standard web scraping extraction techniques (Villena et al., 2020). It consists of 5, 694 articles published between 2002 and 2020 across 34 journals specialized in health and biology. Descriptive metrics also shown in Table 1.

The algorithm used for the extraction of the journals was constructed using the BeautifulSoup package as follows.

**Algorithm 1:**
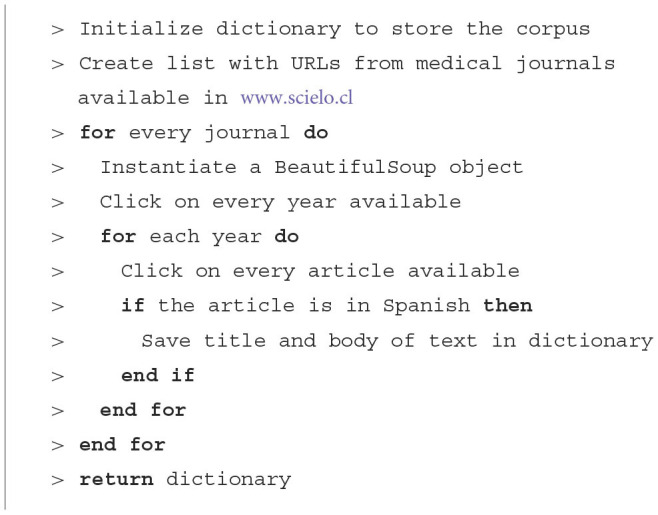
Web scraping extraction.

*UMLS Term Sequences*: This process was inspired by Perozzi et al. (2014) and Tang et al. (2015) who proposed to translate graphs into node sequences to learn network embeddings. The UMLS is a compendium of many controlled vocabularies in the biomedical sciences maintained by the US National Library of Medicine. This vocabulary is hierarchically organized in a graph where each node represents a term from the vocabulary, and each edge represents semantic relationships between these words. We simulate a random walk on the term graph to create a corpus of heading sequences. The full description of this resource is also in Table 1.

The creation of heading sequences works as follows. First, we define the graph *G* = (*V, E*), where *V* corresponds to the set of nodes that in this case represent UMLS terms and *E* is the set of edges between two nodes that are semantically related. As previously stated, *u, v*∈*E* if *u* and *v* are semantically related terms. Then, let *c* be a random walk be such that it starts at node *u* (*c*_0_ = *u*), and let *c*_*i*−2_ = *t*, *c*_*i*−1_ = *v* and *c*_*i*_ = *x* be three continuous nodes in the chain, *t, v, x*. The distribution generated for *c*_*i*_ is defined as:


$$\mathbb{P}(c_{i}=x|c_{i-1}=v)=\{\begin{array}{cc} \pi_{vx} & \text{if }(v,x)\in E,\\ 0 & \text{in another case,} \end{array}$$

where π_*vx*_ is the transition matrix from *v* to *x*:


$$\pi_{vx}=\alpha (t,x)=\{\begin{array}{cc} \frac{1}{p} & \text{if }d_{tx}=0,\\ 1 & \text{if }d_{tx}=1,\\ \frac{1}{q} & \text{if }d_{tx}=2, \end{array}$$

where *d*_*tx*_∈{0, 1, 2} is the shortest path between *t* and *x* (it takes values 0, 1, or 2 because the nodes *t, v, x* are consecutive on the chain). We used *p* = 2 and *q* = 1 following the work by Zhang et al. (2019). This random walk was implemented using the node2vec package.

The Chilean waiting list, the medical journals and the UMLS heading sequences are all word sequences, the fastText model (which works with subword information) can share the n-gram representations between all three corpora, thus consolidating them into a single corpus. This corpus was preprocessed by transforming each character to lowercase, removing character accentuation and removing non-alphabetic symbols. Finally, the corpus was tokenized by sentence, and each sentence was tokenized by word.

In sum, we have worked with a corpus of 235 million tokens, which is a good number compared to, for example, the 86 million tokens collected by Akhtyamova et al. (2020). Nevertheless, our corpus is still way below the 13.5 billion tokes used to calculate BERT for the clinical domain in English (Lee et al., 2020).

### 3.2. Embeddings computation

Word2vec and fastText are two of the most used algorithms for the computation of static word embedding language representations. Both algorithms use the same principle, where a shallow artificial neural network is trained to predict the context of a certain token in a sentence (skip-gram algorithm) or to predict a token given a set of context tokens (continuous bag of words algorithm), and then, use the trained parameters representing each word as the output of the algorithm.

The fastText algorithm uses subword unit combinations, learning embeddings for *n*-grams of the characters of each word. Then, if this method encounters an unknown word, its embeddings will be induced by the average vector representation of its constituent *n*-grams. This presents a straightforward solution to the out-of-vocabulary (OOV) problem.

The embeddings are computed by standard skip-gram models considering the subword information given by character n-grams. Another critical component in this formulation is the position-dependent weighting of the words: the model learns position representations and uses them to reweight the vectors using linear combinations of both encoding and position of the word.

We chose to work with fastText because, as seen in Zhang et al. (2019), the Word2vec algorithm is only based on unigrams, and therefore cannot recognize OOV words. However, we still trained and used a Word2vec model to perform the other validation tasks (see Section 4). Word2vec is one of the most used algorithms when it comes to word embeddings. It allows an accurate vector representation of words which can be computed on the CPU without the need for high computing power or special computing modules (Mikolov et al., 2013). We first decided to train this model with our corpus merely as a baseline, but were surprised by the good results in the experiments despite the lack of subword information.

### 3.3. Training

As seen in Zhang et al. (2019) and Soares et al. (2019), fastText computes embeddings by encoding every word through calculation of the sum vector of its constituent character n-grams. The positioning weight was calculated using a standard skip-gram model with a window size of 20. This number was chosen by creating different models (Word2vec and fastText) with different window sizes ranging from 5 to 25 words. Each model was tested with the validation tasks (see Section 4), and the model with the best performance was kept. This idea was first suggested by Chiu et al. (2016), who stated that intrinsic tasks benefit from bigger window sizes. The setup used for both Word2vec and fastText was the default (skip-gram) for each implementation except for the vector dimension, which was set to 300.

We computed the vector representations using the original algorithm implementations proposed by the authors of Word2vec^6^ and fastText^7^. Due to the large amount of data used to compute the representations we utilized two nodes of 20 CPU cores from a High-Performance Computing Cluster. The word embeddings can be download from Zenodo (see text footnote 1).

### 3.4. Validation dataset

Intrinsic evaluation tests the quality of syntactic or semantic relationships between words. There exist tasks for intrinsic evaluation of a clinical word embedding model in Spanish, for example, the UMNSRS-Sim and UMNSRS-Rel databases for word pair similarity developed by Pakhomov et al. (2010) and translated by Soares et al. (2019). The original dataset consists of pairs of UMLS concepts manually annotated for similarity and relatedness.

There is an intrinsic evaluation task called sentence pair similarity, which, to the best of our knowledge, has not been translated to Spanish before. To implement this task, we used the BioCreative/OHNLP STS 2019 from Wang et al. (2018, 2020), which contains 1, 643 sentence pairs, alongside their similarity score provided by a group of experts on a scale from 0 to 5. Here, 0 means the sentences are completely different, and 5 means they could be used interchangeably. The Mayo Clinic developed this dataset in the context of their SemEval Semantic Textual Similarity (SemEval STS) challenge^8^.

The translation of these sentences can be challenging since translating sentences from a specialized domain is not as straightforward as translating single words, as was cleverly done by Soares et al. (2019) using bilingual UMLS. Our validation of the translation was done in two stages, having as input the 1, 643 sentence pairs translated from English to Spanish using Google Cloud Translation API^9^.

A random subset of 100 of these sentences was reviewed by two bilingual domain specialists, who identified problematic cases in each translation. A translation standard was established to identify problematic cases based on three types of inadequacies. This validation standard was designed for this specific task and inspired by the work of Castillo-Orueta et al. (2018) and Ortiz-Gutiérrez and Cruz-Avelar (2018). Thus, each possible problematic case corresponds to a lexicalization that has presented at least one of these three inadequacies:

- Terminological inadequacy: the lexicalization in the target language does not capture the technicality of the concept in the source language (e.g., the medication *concerta* translated as *concierto*).
- Grammatical inadequacy: the lexicalization in the target language is incorrect in terms of the grammatical expressions of the source language (e.g., *All the patient's questions were answered to the best of my ability*. translated as *Todas las preguntas del paciente fueron respondidas lo mejor que pueden*).
- Functional inadequacy: the lexicalization in the target language is inappropriate to the register or style of the source language (e.g., *by mouth* translated as *via de la boca*).

**Figure 1:**
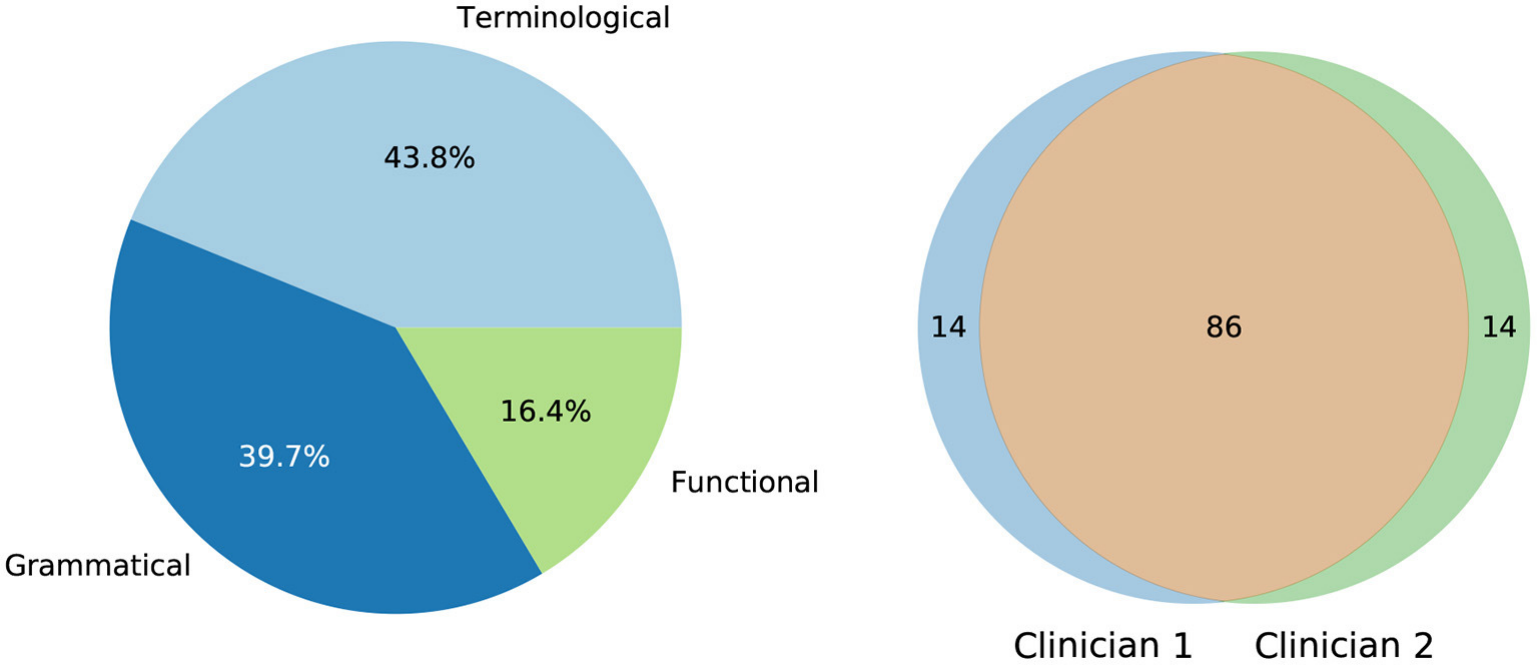
**(Left)** Distribution of types of specialist-identified translation inadequacies among target language lexicalizations. **(Right)** Intersection of agreed inadequacy types between both specialists. Each number represents the size of the set.

The inadequacy detection procedure achieved almost perfect agreement between specialists (Cohen's kappa coefficient of 0.86). In the 14 cases where the domain experts had discrepancies, the research team discussed the cases and took a decision, creating a consolidated validation.Out of these 100 sentences, 31 sentences were classified correct, 30 contained at least one terminological inadequacy, 30 contained at least one grammatical inadequacy, and 9 contained at least one functional inadequacy. If a sentence contained multiple types of inadequacies, it was classified under the most severe type of inadequacy present according to the order in which they were listed above, i.e., terminological—grammatical—functional. The distribution of these types of inadequacies and the agreement between specialists is visualized in Figure 1.

The high occurrence of translation errors reveals the shortcomings of generalized machine translation models when applied to tasks involving specialized language. While Google Cloud Translation API displays robust performance for generalized language use cases, it produces many translation errors when applied on clinical language, some of which can produce critical misunderstandings due to the precise nature of the subject. This implies the necessity of fine-tuned models for specialized tasks such as clinical translation. Additionally, since the clinical text is normally written with stress and time constraints, the original English sentences are not entirely error-free; such errors can carry over in the automatic translation. For example, the missing apostrophe in *patients symptoms* lead to the translation *pacientes*.

To perform the word embedding validation using this dataset, the vectors of each word constituting the sentence were averaged. Then, the cosine similarity, Euclidean, and Manhattan distance were calculated. These are the same scores used in the SemEval STS challenge, allowing us to compare the results. We cannot openly share this validated dataset since it is a translation of the original dataset that is not public and only available by request to the authors of Wang et al. (2018, 2020).

## 4. Results

### 4.1. Word pair similarity

As mentioned above, for the word pair similarity task, we used the translated versions of UMNSRS-Sim and UMNSRS-Rel. The results for the former are shown in Table 2, where we compare our model with the state-of-the-art provided by Zhang et al. (2019), BioWordVec, and the Spanish model by Soares et al. (2019) SHE (Spanish Health Embeddings). For our embeddings, the corpus called *Mix* refers to a merging of the Chilean Waiting List, the medical journals, and the UMLS heading sequences corpora.

**Table 2:** Comparison between proposed embeddings (ours), the state-of-the-art in English (BioWordVec), and a similar model in Spanish (SHE) using the UMNSRS-Sim set.

**Embedding**	**Corpus**	**Pairs evaluated**	**Pearson**
BioWordVec	PubMed + MeSH	521	**0.665**
SHE	SciELO	322	0.582
Ours (Word2vec)	Mix	268	0.583
Ours (fastText)	Mix	286	0.588

Bold values correspond to the best score out of all compared models.

As shown in Table 2, our model was evaluated with roughly half of the original pairs. This is because the pairs of concepts in which one of the terms had a composed word was deleted. In the particular case of Word2vec, the out-of-vocabulary words were also deleted, since this model is unable to recognize them, unlike fastText, which uses sub-words information to create the embeddings, therefore allowing it to represent out-of-vocabulary words.

### 4.2. Sentence pair similarity

To compare with the state-of-the-art results, we measure similarity using cosine, Euclidean, and Manhattan (or Block) distance for the sentence pair similarity task. Then, the calculated score is compared with the referential one using the Pearson correlation. Once again, the results in English prove to obtain higher scores since the task was evaluated in the original language it was designed.

## 5. Discussion

For the first task, sentence pair similarity, we see in Table 3 that both of our models reach similar results as the Spanish embeddings (SHE). The model in English reaches better results because the dataset was initially in this language. Even though the BioWordVec scores are higher, our models are not too far off, despite having fewer pairs of words to compare. As previously discussed, some percentage of similarity between words might be lost in translation.

**Table 3:** Comparison between proposed embeddings (ours), and the state-of-the-art model (BioWordVec) for the sentence pair similarity task.

**Embedding**	**Cosine**	**Euclidean**	**Manthattan**
BioWordVec	0.771	0.753	0.752
Ours	0.642	0.608	0.607

The scores stand for the Pearson correlation between proposed embeddings and referential score.

As for the second task, it was expected for our model to underperform compared to BioWordVec, since the translation of the sentences could have introduced errors. Besides, there is again the issue of scoring discrepancy, since we do not know with certainty if the same level of similarity holds for a pair of translated sentences as it did in the original language.

Our proposal is based on using Word2vec and fastText algorithms to compute static word embeddings, which are single-word representations and cannot account for word polysemy. Nowadays, there is a strong development of contextualized word embeddings that assign dynamic representations to words based on their contexts, achieving state-of-the-art performance in multiple tasks. For the clinical domain in Spanish, relevant works include (Akhtyamova et al., 2020; Carrino et al., 2022; Rojas et al., 2022). These contextualized word embeddings are challenging to compute and deploy in production environments due to their demanding infrastructure needs. Usually, a system based on these embeddings requires costly computation modules to achieve moderate performance. Hospitals in developing countries could benefit from systems based on neural architectures, but the deploying cost might be prohibitive; therefore, solutions based on lightweight methods such as our static word embeddings have the most potential to impact developing societies.

## 6. Conclusion

There is a need to create numerical representations of clinical narratives and support decision-making, especially for languages other than English. In this work, we gathered a corpus for the Spanish language combining clinical text, biomedical journals, and synthetic sentences. This resulted in a rich corpus used to train word embeddings using Word2vec and fastText models. Evaluating these representations within the clinical context is fundamental, and here, we presented tasks adapted from the English language. Our results are not far off from the state-of-the-art for the English language, which is remarkable considering the size of the training corpora, the differences in syntactic structure in English and Spanish, and possible loss in translation upon adapting the tasks.

Given the recent advances in processing power, there is a growing interest within the NLP research community in developing new language models for obtaining contextualized embeddings. However, static embeddings, such as ours, can be used in clinical contexts with limited computational resources and training corpora, which is the situation in many places worldwide. An accurate vector representation of clinical narratives can improve several tasks, such as named entity recognition, text classification, and automatic coding, among many others.

Our group has experience using static word embeddings for patient classification deployed in a hospital (Villena et al., 2021b) and using stacked embeddings that combine both static and contextualized embeddings for named entity recognition (Báez et al., 2020; Báez et al., 2022) and automatic coding (Villena et al., 2021a). Evaluating the automatic translation of clinical sentences has pointed us the need for creating reliable intrinsic tests created from scratch for the Spanish language, which can be valuable for both static and contextual word embeddings. An even more ambitious challenge is to work with clinicians for different Spanish-speaking countries to make this evaluation dataset more robust.

## Data availability statement

The original contributions presented in the study are publicly available. The training corpus and the embeddings can be downloaded from https://zenodo.org/record/6647060#.Yw_j4ezMI-R.

## Author contributions

FV, JD, CC, and FN contributed to the conception and design of the study. CC and FV created the corpus and trained word embeddings. FN designed the validation standard for the translation. FV and CB typify inadequacies. FN, KM, JD, and CC decided on the validations with discrepancies. CC wrote the first draft of the manuscript. CC, FV, KM, FN, and JD wrote sections of the manuscript. All authors contributed to manuscript revision, read, and approved the submitted version.

## Funding

This work was funded by ANID Chile: Basal Funds for Center of Excellence FB210005 (CMM), Millennium Science Initiative Program ICN2021_004 (iHealth), and Fondecyt grants 11201250 and 1210648. This research was partially supported by the supercomputing infrastructure of the NLHPC (ECM-02) and the Patagón supercomputer of Universidad Austral de Chile (FONDEQUIP EQM180042). The MISTI Chile program of MIT funded KM's travel.

## Conflict of interest

The authors declare that the research was conducted in the absence of any commercial or financial relationships that could be construed as a potential conflict of interest.

## Publisher's note

All claims expressed in this article are solely those of the authors and do not necessarily represent those of their affiliated organizations, or those of the publisher, the editors and the reviewers. Any product that may be evaluated in this article, or claim that may be made by its manufacturer, is not guaranteed or endorsed by the publisher.

## Footnotes

^2^ https://zenodo.org/record/6647060#.YqoiSuzMI-Q

^3^ https://crscardellino.github.io/SBWCE/

^4^ https://paperswithcode.com/dataset/chilean-waiting-list-corpus

^5^ https://doi.org/10.5281/zenodo.5902835

^6^ https://github.com/tmikolov/word2vec

^7^ https://github.com/facebookresearch/fastText

^8^ https://sites.google.com/view/ohnlp2018/home

^9^ https://cloud.google.com/translate
